# EVI1 in Leukemia and Solid Tumors

**DOI:** 10.3390/cancers12092667

**Published:** 2020-09-18

**Authors:** Beiyuan Liang, Jing Wang

**Affiliations:** Department of Cancer Biology and Genetics, College of Medicine, The Ohio State University, Columbus, OH 43210, USA; liang.985@osu.edu

**Keywords:** EVI1, MDS1/EVI1, AML1/MDS1/EVI1, leukemia, transcription factors, cancer, solid tumors

## Abstract

**Simple Summary:**

*Ecotropic viral integration site 1 (EVI1)* is transcriptionally activated in a subset of myeloid leukemias. Since its discovery, other isoforms of EVI1 have been identified. It has been shown that EVI1 and its isoforms mainly function as transcription factors and to play important roles not only in leukemia but also in a variety of solid tumors. To provide a comprehensive understanding of this family of proteins, we summarize the currently available knowledge of expression and function of EVI1 and its isoforms in leukemia and solid tumors and provide insights of future studies.

**Abstract:**

The *EVI1* gene encodes for a transcription factor with two zinc finger domains and is transcriptionally activated in a subset of myeloid leukemias. In leukemia, the transcriptional activation of *EVI1* usually results from chromosomal rearrangements. Besides leukemia, *EVI1* has also been linked to solid tumors including breast cancer, lung cancer, ovarian cancer and colon cancer. The *MDS1/EVI1* gene is encoded by the same locus as *EVI1*. While EVI1 functions as a transcription repressor, MDS1/EVI1 acts as a transcription activator. The fusion protein encoded by the *AML1/MDS1/EVI1* chimeric gene, resulting from chromosomal translocations in a subset of chronic myeloid leukemia, exhibits a similar function to EVI1. EVI1 has been shown to regulate cell proliferation, differentiation and apoptosis, whereas the functions of MDS1/EVI1 and AML1/MDS1/EVI1 remain elusive. In this review, we summarize the genetic structures, biochemical properties and biological functions of these proteins in cancer.

## 1. Introduction

Ecotropic viral integration site 1 (EVI1) is an oncogenic transcription factor that plays an important role in development and oncogenesis. *EVI1* normally exists as a single gene, or as a longer form in a fusion gene with *myelodysplastic syndrome 1 (MDS1)*, called *MDS1/EVI1* [1]. In a subset of chronic myeloid leukemia (CML) associated with t(3;21), *EVI1* also exists as a chimeric fusion gene called *AML1/MDS1/EVI1* [2,3].

Studies have shown that both EVI1 and AML1/MDS1/EVI1 mainly function as a transcription repressor, whereas MDS1/EVI1 functions as a transcription activator [4,5,6,7]. *EVI1* is overexpressed in about 10% of adult acute myeloid leukemia (AML) and acts as a marker in myeloid malignancies for an aggressive disease with poor survival [8,9,10,11,12,13,14,15,16,17]. EVI1, MDS1/EVI1 and AML1/MDS1/EVI1 promote leukemogenesis by modulating differentiation, apoptosis, cell cycle and proliferation. *EVI1* expression is not only upregulated in leukemia, but also in a number of solid tumors, including colorectal cancer, breast cancer, prostate cancer and ovarian cancer [18,19,20,21,22,23,24,25,26]. *EVI1* has been shown to be redistributed or overexpressed in several types of solid tumors. Higher levels of *EVI1* messenger RNA (mRNA) were detected in ovarian carcinoma compared to a normal ovary [25]. Similarly, EVI1 also exhibits a redistribution of expression in prostate cancer. In healthy prostatic tissues, *EVI1* is expressed in the prostate stem cell compartment located at the basal layer. However, in a prostate cancer progression cohort consisting of 219 samples from patients with primary prostate cancer, lymph nodes and distant metastases, *EVI1* was found to be heterogeneously distributed within samples [26]. Expression of EVI1 is low in adult normal small intestine and colon. However, previous studies on *EVI1* in colorectal cancer have revealed that expression of *EVI1* was upregulated in colon cancer patient samples and colon cancer cell lines, such as HCT116 cells and HT-29 cells [20,21]. Functionally, *EVI1* modulates cell proliferation, cell cycle progression, migration and apoptosis. Several studies have shown that a number of signaling pathways are regulated by *EVI1*, including the epithelial-mesenchymal transition (EMT)-related, phosphatase and tensin homolog (PTEN)/AKT/the mammalian target of rapamycin (mTOR), transforming growth factor (TGF)-β signaling, apoptosis and cell cycle-related pathways [27,28,29,30,31,32,33,34,35]. Taken together, these studies suggest that upregulation of *EVI1* expression could be a functionally important molecular event during cancer development and progression, and therefore *EVI1* could be a potential molecular target for cancer treatment.

### 1.1. Structure of EVI1 and MDS1/EVI1

**Figure 1:**
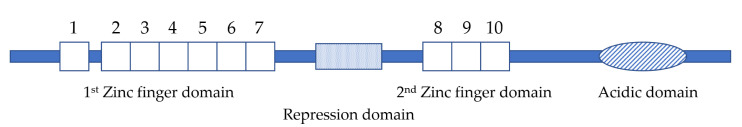
EVI1 protein structure. The numbered boxes indicate individual zinc fingers. The dotted box indicates the proline-rich repression domain. The oval indicates the acidic domain.

*EVI1* was initially identified as a common ecotropic retrovirus integration site in murine leukemia [36]. Subsequent sequencing studies showed that EVI1 contains two DNA binding domains with seven and three repeats of the zinc finger motif, respectively [37]. The first zinc finger domain containing seven zinc finger motifs is located at the amino terminus and the second one consisting of three zinc finger motifs lies towards the carboxyl terminus. Both zinc finger domains bind to specific DNA sequences. The first one recognizes a consensus sequence of GA(C/T)AAGA(T/C)AAGATAA [38], whereas the second one binds to a consensus sequence of GAAGATGAG [39]. A highly acidic domain is also found near the carboxyl terminus of EVI1. Although acidic domains are often found in transcription factors as part of the transcriptional activation domains, no data have shown that the acidic domain of EVI1 is essential for transcriptional activation [39]. In addition, transcriptional repressor domains are also identified in EVI1, such as the proline-rich repression domain [39] (Figure 1). Both human and murine *EVI1* complementary DNAs (cDNA) share more than 90% homology [40].

*MDS1* was first identified in a fusion gene resulting from chromosomal rearrangements involving chromosome band 3q26. This 3;21 translocation leads to the expression of several fusion proteins including AML1/MDS1, AML1/EAP and AML1/MDS1/EVI1 [1,2,41,42]. The *MDS1* locus is mapped 150–300 kb upstream of *EVI1* at chromosome band 3q26 [2].

**Figure 2:**
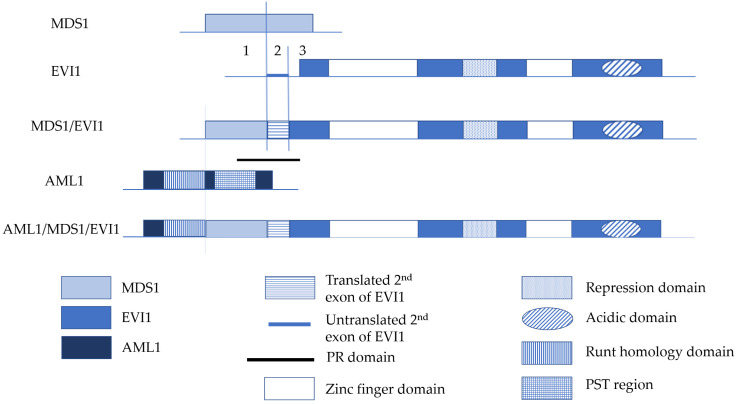
Diagram illustrating the relationship between *EVI1*, *MDS1/EVI1* and *AML1/MDS1/EVI1*. Untranslated regions are indicated by lines and reading frames are indicated by boxes. The vertical lines in MDS1/EVI1 indicate the junction with the 3′ alternative coding exon in MDS1, as well as the junctions between exon 1, 2 and 3 in EVI1. Numbers indicate EVI1 exons. The black thick line indicates the position of the PR domain. The vertical line in AML1/MDS1/EVI1 indicates the junction between AML1 and MDS1/EVI1, which is at the end of the runt homology domain.

Fears et al. [1] performed Northern blot in multiple tissues with an *MDS1* probe and showed two large bands of 5.8 and 6.2 kb, which were identical to those observed with an *EVI1* probe, suggesting that *MDS1* and *EVI1* could be expressed as one transcript, designated as *MDS1/EVI1*, in normal tissues. The existence of the *MDS1/EVI1* transcript was confirmed by isolating and sequencing cDNA clones from normal human pancreas and kidney libraries. Sequence analysis of the clones showed an open reading frame in which *MDS1* was spliced in frame to the second exon of *EVI1*, resulting in a new gene, *MDS1/EVI1* [1]. In EVI1, the first methionine is located in the third exon. In *MDS1/EVI1*, the splicing site is at the junction between exon 1 and exon 2 of *EVI1* [42]. With 125 codons from *MDS1* and 63 codons from the second and part of the third exon of *EVI1*, a total of 188 codons are added upstream of the start codon of *EVI1* [42] (Figure 2). This new *N*-terminal extension of EVI1 has later been named the PR domain [1] (Figure 2), and shares about 40% homology with PR domains found in several proteins including *C. elegans* differentiation factor egl-43 [43], B cell factor positive regulatory domain I binding factor 1 (PRDI-BF1) [44,45,46] and retinoblastoma binding protein RIZ [47,48]. The homology between MDS1/EVI1 and egl-43 extends from PR domains to one of the zinc finger domains [43,49]. Experiments in *C. elegans* and in mice where egl-43 and MDS1/EVI1 were disrupted by homologous recombination showed that disruption of either protein prohibits the development of the nervous system [43,49], further suggesting the close relationship between these two proteins.

AML1 contains a runt homology domain (RHD) at the *N*-terminus which is highly homologous to the *Drosophila melanogaster* segmentation gene *runt* and a subunit of polyomavirus enhancer binding protein 2 (PEBP2 or PAE2). A proline-serine-threonine (PST) domain is located at the C-terminus of AML1 which is required for transcriptional activation [50]. *AML1/MDS1/EVI1* contains an open reading frame of 4185 nucleotides and encodes a 1395 amino acid protein [3]. In *AML1/MDS1/EVI1*, *AML1* is interrupted at the end of RHD, followed by the entire *MDS1/EVI1* cDNA. Therefore, the AML1/MDS1/EVI1 fusion protein is a chimeric protein which consists of the RHD of AML1, the PR domain and two zinc finger domains of MDS1/EVI1 (Figure 2).

Besides MDS1/EVI1, EVI1 also has a few alternative splice transcripts which encode for truncated forms of EVI1, called EVI-Δ324 and EVI1-Δ105. Although some studies have revealed part of the function of EVI1 and MDS1/EVI1, little is known about the function of the splicing variants of EVI1.

### 1.2. Expression and Function of EVI1 and MDS1/EVI1 in Development

Perkins et al., 1991, first reported an expression pattern of EVI1 in embryonic and adult mouse tissues. They showed that *EVI1* expression was high in lung, heart, urinary system and Mullerian ducts. Additionally, *EVI1* was detectable in primary fetal cells, differentiating red blood cells, developing limbs and developing oocytes in the ovary [51]. The spatial and temporal expression pattern of EVI1 suggests that *EVI1* plays an important role in mouse development. However, these studies by Northern blots were carried out with a probe that also recognizes *MDS1/EVI1*. Thus, it is unknown whether the results are specific for *EVI1* or *MDS1/EVI1* or both. Later on, Nucifora et al. [2] showed, by Northern blot with probes specific for *MDS1/EVI1*, that *MDS1/EVI1* is expressed at high levels in adult kidney, pancreas and lung, but at lower levels in several other adult tissues including heart, placenta and skeletal muscle. However, MDS1/EVI1 was not detectable in thymus, spleen, leukocytes, peripheral blood or myeloid [2].

More studies carried out by Sitailo et al. [52] showed that expression of *EVI1* and *MDS1/EVI1* is regulated independently during embryonic stem cell differentiation. *EVI1* was not detectable in undifferentiated totipotent embryonic stem cells. During embryonic stem cell differentiation, *EVI1* appears on day 3 and disappears on day 13. MDS1/EVI1 is expressed at low levels in undifferentiated totipotent embryonic stem cells. Expression of *MDS1/EVI1* is at the highest on day 7 of differentiation and persists for about 14 days [52].

Bartholomew et al. [4] showed for the first time that EVI1 functions as a transcription repressor. They used deletion mutagenesis to show that the repressor activity of EVI1 lies between amino acids 514 and 714 [4]. Fusion proteins lacking this region retain nuclear localization, indicating that loss of transcriptional repression activity is not due to changes in subcellular localization [4]. Soderholm et al. [5] compared the transcriptional activity of MDS1/EVI1 and EVI1 and showed that MDS1/EVI1 and EVI1 both recognize and bind to the consensus sequence of the first zinc finger domain. By using deletion mutants, they demonstrated that the binding was due to the proximal region of zinc fingers [5]. Using reporter assays in which CAT gene expression was controlled by a 207 bp genomic promoter with nine AGATA repeats, they also showed that activation of the promoter by MDS1/EVI1 was repressed by EVI1 [5]. In addition, another group demonstrated that MDS1/EVI1 increased GATA-binding factor 1 (GATA-1) transcriptional activity whereas EVI1 acted as a repressor of GATA-1 [53]. Therefore, removal of the *N*-terminal extension of 188 amino acids converts MDS1/EVI1 from a transcriptional activator to a repressor. Further work indicated that the PR domain, which is absent in EVI1, is responsible for the activating properties of MDS1/EVI1, because it functions as a transcriptional activation domain when fused with the DNA binding domain of Gal-4 [5]. Taken together, EVI1 and MDS1/EVI1 are two different proteins with large overlaps. While EVI1 is a transcriptional repressor, MDS1/EVI1 is a strong transcriptional activator.

EVI1 is involved in cell proliferation, vascularization and differentiation [49]. The full-length *EVI1* transcript was disrupted by targeted mutagenesis in embryonic stem cells and showed that embryos with the EVI1 homozygous mutant died on day 10.5. At that time, mutant embryos were easily differentiated from wild type embryos by hemorrhaging, widespread hypocellularity and failure in the development of paraxial mesenchyme [49]. Additionally, the peripheral nervous system failed to develop in mutant embryos and defects in somites, heart and cranial ganglia were detected [49]. The defects in cellular proliferation observed in mutant embryos indicate that EVI1 is involved in the regulation of cell growth. A wide range of defects agree with the expression pattern of EVI1 during embryonic development [51]. However, at the time of the study, the existence of *MDS1/EVI1* was unknown. Therefore, this study only demonstrates the importance of the gene locus shared by *EVI1* and *MDS1/EVI1* during embryonic development.

In an attempt to differentiate *EVI1* and *MDS1/EVI1* function, Zhang et al. [54] generated a mouse model in which *MDS1/EVI1* expression was eliminated while expression of *EVI1* remained unchanged. This study and others demonstrate that *EVI1* and *MDS1/EVI1* may exert their functions in modulating hematopoiesis at different stages and by different mechanisms [9,54].

Taken together, these results suggest that MDS1/EVI1 is expressed in normal tissues rather than EVI1, and that the inappropriate expression of EVI1, an altered activator which has become a repressor, could be the cause of leukemic transformation [5]. Although the expression and function of *MDS1/EVI1* in malignancy remain elusive, EVI1 has been extensively studied in cancer, especially leukemia.

### 1.3. EVI1 in Myeloid Leukemias

EVI1 has been established as a marker in myeloid leukemias for aggressive disease with poor survival [8,10,11,12,13,55]. Chromosomal rearrangements involving 3q with EVI1 overexpression are found in 2–2.5% AML cases, whereas EVI1 overexpression is seen in 6–11% of adult AML [14,15,16,17]. Both inv(3)/t(3;3) and EVI1 overexpression alone are well-established prognostic markers in AML associated with poor outcomes [15,16,17]. In the most recent 2008 World Health Organization (WHO) classification, AML with inv(3)/t(3;3)(q21q26) has been categorized as a distinct entity characterized by its aggressive course and poor prognosis [56]. In a study of 319 de novo AML patients, high expression of EVI1 but not MDS1/EVI1 was associated with highly aggressive AML [15]. In another study of 266 AML patients, it was shown that both EVI1 and MDS1/EVI1 overexpression predict short remission duration [57]. In CML, EVI1 overexpression has been associated with the higher self-renewal capacity of CML stem cells and resistance to tyrosine kinase inhibitors in murine models [11]. The prognostic value of AML1/MDS1/EVI1 in leukemia has yet to be determined.

### 1.4. Chromosomal Rearrangements Activate EVI1 Transcription in Leukemia

EVI1 expression is not detectable in the bone marrow of normal individuals. However, *EVI1* transcription has been found to be inappropriately activated in mouse and human hematopoietic cells of myeloid leukemia [58]. In human leukemia, activation of *EVI1* transcription is usually caused by chromosomal rearrangements at the site of the *EVI1* locus, 3q26 [59,60], among which, the most frequent involve 3q21 and 3q26, resulting in t(3;3)(q21q26) and inv(3)(q21q26). Using pulsed field gel electrophoresis (PFGE) and fluorescence in situ hybridization (FISH), the breakpoints of t(3;3) have been mapped at the 5′ end of *EVI1*, whereas the breakpoints of inv(3) have been mapped at the 3′ end [61,62]. Due to the specific orientation of the two breakpoints on chromosome 3, it was previously speculated that the activation may result from juxtaposition of *EVI1* to the enhancer of *Ribophorin I*, a constitutively expressed housekeeping gene, to the coding region of *EVI1* [62]. Later, it was shown that translocation of a *GATA2* enhancer to 3q26 results in overexpression of *EVI1* [63,64].

Activation of *EVI1* transcription can also result from translocations involving chromosomes other than 3, leading to the constitutive expression of a fusion protein in which the entire EVI1 locates at the carboxyl end. The most frequent cases are t(3;21)(q26;q22) and t(3;12)(q26;p13) [2,3,65], which are seen in myelodysplastic syndromes (MDS), AML or CML during the blast crises [66,67,68,69]. t(3;21) results in fusion between the DNA binding domain of AML1 and MDS1/EVI1 [2,3], whereas t(3;12) leads to the fusion between the amino terminus of the ETS protein TEL to MDS1/EVI1 [70,71,72]. While AML1 and TEL are related to a large number of frequent chromosomal rearrangements in myeloid and lymphoid leukemias [73,74,75], EVI1 has been associated with chromosomal rearrangements in myeloid leukemias exclusively. Both *AML1/MDS1/EVI1* and *TEL/MDS1/EVI1* are transcribed by the promoter of *AML1* or *TEL*.

## 2. EVI1, MDS1/EVI1 and AML1/MDS1/EVI1 in Leukemogenesis

### 2.1. EVI1 and MDS1/EVI1 in Leukemogenesis

#### 2.1.1. Differentiation

Overexpression of EVI1 prevented 32Dcl3 cells from expressing myeloperoxidase and differentiating to granulocytes after granulocyte colony stimulating factor (G-CSF) treatment [52]. Later Khanna-Gupta et al. [76] showed that 32Dcl3 cells contain a rearrangement at the *EVI1* locus and constitutively overexpress EVI1. EVI1 expression decreased only slightly during G-CSF-induced myeloid maturation. Activation of EVI1 transcription in naive 32Dcl3 cells possibly contributes to the immortalization of the cell line by impairing spontaneous differentiation. These results do not conflict with the observation from Sitailo et al. [52] that overexpression of EVI1 blocks differentiation in 32Dcl3 cells in response to G-CSF, but rather suggest that higher levels of EVI1 are required to block G-CSF-induced differentiation than spontaneous differentiation in myeloid cells [52].

Overexpression of EVI1 in primary hematopoietic cells from mouse bone marrow decreased their ability to form burst-forming units-erythroid (BFU-E) in semisolid medium [53]. Thus, a potential mechanism for the leukemogenic and myelodysplastic effects of *EVI1* is the inhibition of the differentiation of hematopoietic cells. Louz et al. [77] showed that disruption of erythropoiesis is seen in one of three mouse lines of transgenic mice overexpressing *EVI1*.

Glass et al. [78] conducted a comprehensive genome-wide study of EVI1 DNA binding sites in leukemic cells and compared whole transcriptome gene expression profiles between EVI1-overexpressed and EVI1 knockdown leukemic cells using chromatin immunoprecipitation-Sequencing (ChIP-Seq) and RNA-Sequencing (RNA-Seq) expression profiling. They showed that EVI1 can directly bind to and downregulate the master myeloid differentiation gene, *Cebpe*. Previous studies have shown that *Cebpe* plays a critical role in the terminal differentiation of granulocytes [79,80,81]. In two different *EVI1*-overexpressing leukemic cell lines, DA-1 cells and NFS-60 cells, *Cebpe* was found to be downregulated by 2-fold. Several *Cebpe* downstream target genes, including *Epx*, *Lcn2*, *Mmp8* and *Prg2*, were also significantly downregulated in both EVI1 leukemic cells [78].

#### 2.1.2. Apoptosis

Besides terminal myeloid differentiation, Glass et al. [78] showed that EVI1 target genes are also related to apoptosis. They identified seven significantly downregulated genes that encode for ligand gated p2 purinoreceptors, including *P2rx3*, *Prx4*, and *P2rx7*. They showed that EVI1 binds to three different sites within the *P2rx7* promoter region and significantly downregulates *P2rx7* transcription in AML [78]. P2RX7 is a cell surface ATP receptor mainly expressed in macrophages and neutrophils and mediates ATP-induced apoptosis of macrophages and neutrophils. It has been shown that loss of function of the P2RX7 receptor impaired apoptosis [82,83,84,85]. Activation of the P2RX7 receptor leads to activation of caspase-1 [86]. Humphreys et al. [87] showed that P2RX7 stimulation in response to ATP rapidly increases caspase-3 protease activity, which is associated with DNA fragmentation and upregulation of the c-Jun *N*-terminal kinase pathway [87].

Perkins et al. [88] investigated the physiological effects of *EVI1* knockdown in murine leukemic cells expressing EVI1. They showed that knockdown of EVI1 induces apoptosis via the intrinsic pathway but not the extrinsic pathway. Specifically, procaspase 3 and 9 were cleaved but caspase 8 or Bid remained unchanged. DNA fragmentation and histone release were both induced, and mitochondrial membrane potential was reduced.

#### 2.1.3. Cell Quiescence

Zhang et al. [54] generated an MDS1/EVI1 knockout mouse model in which hematopoietic stem cells (HSC) had a high proliferation rate, indicating HSCs are shifted from quiescence to active cycling, leading to a reduction in the number of HSCs and a loss of long-term repopulation capacity. RNA sequencing analysis in HSCs of MDS1/EVI1-KO mice revealed a significant decrease in the expression of *Cdkn1c*, an important negative growth regulator that encodes for p57-Kip2, which preferentially inhibits cyclin E-Cdk2, a G1 cyclin complex. Reintroduction of MDS1/EVI1 into the HSCs normalizes both low-expression p57-Kip2 and the high level of cell proliferation [54]. However, the mechanism by which MDS1/EVI1 regulates *Cdkn1c* transcription has not been elucidated.

To further investigate the effects of *EVI1* overexpression in HSCs, Glass et al. [89] generated a mouse model in which EVI1 can be induced in the HSC compartment. In this model, supplementation of doxycycline can lead to an upregulation of *EVI1* transcripts of 10,000-fold, whereas *MDS1/EVI1* transcripts remain unchanged. The upregulation of EVI1 caused cell cycle arrest in HSCs [89]. Kustikova et al. [90] used a Rosa26rtTA-nls-Neo2 mouse model and overexpressed EVI1 in hematopoietic progenitor cells. They showed that inducible expression of EVI1 leads to cell cycle arrest in G0/G1 in hematopoietic progenitor cells. Gene expression microarray showed enhanced expression of cell cycle inhibitory genes *Cdkn1b* and *Cdkn1c* and downregulation of cyclins and their kinases [90]. These data suggest that EVI1 may contribute to the enlargement of a population of quiescent hematopoietic stem cells.

Studies by Yamakawa et al. [91] showed that overexpression of EVI1 inhibits cell growth and causes cells to accumulate in the G0 phase in. Konrad et al. [92] showed that induction of either EVI1 or MDS1/EVI1 inhibits cell proliferation by slowing the transit through the cell mitotic cycle. Induction of either protein drives cells to accumulate at the G0/G1 phase and moderately increased the rate of spontaneous apoptosis. Overexpression of EVI1 in U20S cells induced the accumulation of supernumerary centrosomes in cells and resulted in a cell cycle arrest in the G0/G1 phase. In addition, EVI1-overexpressing cells showed higher levels of cyclin D1 and p21, reduced Cdk2 activity and activated p53 pathway [93,94]. These data suggest that EVI1 may play a role in cell quiescence.

### 2.2. AML1/MDS1/EVI1 in Leukemogenesis

#### 2.2.1. Repression of TGF-β-Mediated Growth Inhibition

AML1/MDS1/EVI1 inhibits transactivation of TGF-β-responsive promoters similar to EVI1. Expression of EVI1 or AML1/MDS1/EVI1 in 32Dcl3 cells overcomes TGF-β-mediated cell growth inhibition. AML1/MDS1/EVI1 can physically bind to SMAD3 and inhibit its activity to induce transcription of TGF-β target genes. AML1/MDS1/EVI1 also interacts with CtBP through the CtBP-binding consensus sequence PLDLS, which is involved in TGF-β-mediated gene transcription [7].

#### 2.2.2. Stimulation of Proliferation

Kurokawa et al. [95] introduced AML1/MDS1/EVI1 into Rat1 fibroblasts and showed that AML1/MDS1/EVI1 expressing Rat1 cells form macroscopic colonies in soft agar, whereas control cells produce tiny, barely macroscopic colonies, suggesting that AML1/MDS1/EVI1 is a transforming gene. They also demonstrated that introduction of AML1/MDS1/EVI1 into Rat1 clones harboring BCR/ABL conferred a higher capacity for anchorage-independent growth. Deletion mutant analysis showed that removal of the second zinc finger domain of EVI1 completely abrogated the ability of AML1/MDS1/EVI1 to transform Rat1 cells [95]. The transforming effect was due to AP-1 activation by AML1/MDS1/EVI1 [96]. These results suggest that AML1/MDS1/EVI1 could play a pivotal role in the development of chronic myelogenous leukemia [6].

## 3. EVI1 and MDS1/EVI1 in Solid Tumors

Besides leukemia, EVI1 has been linked to other cancers as well. In recent years, it has been shown that *EVI1* is also involved in the occurrence and progression of some solid tumors, including glioblastoma, squamous cell lung cancer, ovarian cancer, prostate cancer and breast cancer. Hou et al. [97] assessed the clinical significance of the MDS1 and EVI1 complex locus protein (MECOM) in glioblastoma multiforme (GBM). They showed that MECOM is highly expressed in 41.9% of GBM tumor samples using immunohistochemistry. In addition, MECOM mRNA expression is also higher in tumor tissues than in normal tissues. They further indicated that higher expression of MECOM was associated with a lower overall survival rate. The one-year survival rate of the MECOM high expression group was three times lower than that in the MECOM low expression group. In addition, they identified MECOM expression as an independent prognosis marker in GBM [97]. Xu et al. [18] showed that 32.32% of squamous cell lung cancer samples express a high level of EVI1. High expression of EVI1 was significantly associated with a poorer five-year survival rate of squamous cell lung cancer patients. Moreover, EVI1 was identified as an independent prognostic factor, suggesting that EVI1 alone was enough to predict poor prognosis of squamous cell lung cancer [18]. Another study done by Wang et al. [19] demonstrated EVI1 overexpression in both estrogen receptor-positive (ER +) and estrogen receptor-negative (ER −) breast carcinomas by analyzing a tissue microarray of 608 breast carcinoma patient specimens. Prognostic relevance of EVI1 overexpression was shown in triple-negative breast carcinoma but not in the HER2-positive breast carcinoma subset [19]. In head and neck squamous cell carcinomas, higher EVI1 expression is associated with a higher rate of lymph node metastasis [98].

Redistribution of EVI1 has also been reported in different types of cancer. Using immunohistochemistry, Brooks et al. [25] showed a relative redistribution of EVI1 from the cytoplasm of normal oocytes to increased nuclear and diffused cytoplasmic localization in ovarian tumors. Higher levels of EVI1 mRNA were also detected in ovarian carcinoma compared to the normal ovary, suggesting EVI1 could play a role in ovarian cancer initiation and progression. Similarly, EVI1 also exhibits a redistribution of expression in prostate cancer. EVI1 is expressed in the stem cell compartment which is located at the basal layer. However, EVI1 was distributed heterogeneously within samples in a prostate cancer progression cohort consisting of 219 samples from patients with primary prostate cancer, lymph node and distant metastases. EVI1 expression is associated with tumor progression, suggesting EVI1 expression may be a driver event in prostate cancer [26].

Functionally, knockdown of EVI1 enhanced sensitivity to apoptosis, and inhibited cell cycle progression, proliferation, migration and anchorage-independent growth in human prostate cancer cells. Interestingly, they also showed that EVI1 is involved in the regulation of stem cell properties. While EVI1 expression was upregulated in experimentally derived docetaxel-resistant prostate cancer cells, knockdown of EVI1 restored the sensitivity to docetaxel in these cells. These data suggest that EVI1 may regulate prostate cancer progression and therapy resistance through mediating stem cell properties [26]. In human breast cancer cells, knockdown of EVI1 inhibited proliferation, tumorigenicity and apoptosis resistance. These effects were rescued by estrogen addition in ER + breast carcinoma cells. In addition, estrogen supplementation restored phosphorylated extracellular signal-regulated kinases (pERK) expression in EVI1 knockdown cells, indicating that EVI1 and estradiol signaling merge on MAPK activation. On the contrary, knockdown of EVI1 had no effect on constitutive ERK activity in HER2-positive breast carcinoma cells. By analyzing the transcriptome of control and EVI1 knockdown MDA-MB-231 cells using gene expression microarrays, G-protein-coupled receptor signaling pathways were identified as the most influenced pathways. Investigation of the direct target genes of *EVI1* revealed that GPR54-ligand *KISS1* was a strong candidate among others. ChIP assays revealed that the *KISS1* promoter is a novel target for EVI1 in breast carcinoma. Thus, EVI1 directly modulates G protein-coupled receptors (GPCRs) signaling by targeting the GPR54 ligand KISS1 at the transcriptional level. In addition, pathways involved in cell cycle control and progression, apoptosis resistance and receptor tyrosine-protein kinase ErbB-2 signaling were also affected by EVI1 [19].

Deng et al. [20] assessed the EVI1 expression level in 15 pairs of human colorectal cancer (CRC) samples and 3 pairs of colon adenocarcinoma samples and their adjacent normal mucosal controls. They showed that EVI1 was overexpressed in 53% of CRC samples (8/15) and 100% of colon adenocarcinoma samples. In addition, they determined the EVI1 protein level in human colon cancer cell lines and revealed that EVI1 was overexpressed in 100% of the CRC cell lines tested (5/5), including Caco2, DLD1, HCT116, HT29 and Lovo. EVI1 represses TGF-β signaling and antagonizes its growth inhibitory effect in colon cancer cells [20]. Liu et al. [21] reported that EVI1 is overexpressed in some colon cancer cell lines including HT29 cells and Caco2 cells. Knockdown of EVI1 by small interfering RNA (siRNA) decreased AKT phosphorylation in HT29 cells and increased their sensitivity to taxol-mediated apoptosis [21]. EVI1 directly binds to the B-cell lymphoma-extra large (Bcl-xL) promoter element via the first zinc finger domain and thus inhibits apoptosis. ChIP assays revealed that EVI1 interacts with the Bcl-xL promoter. Knockdown of EVI1 downregulated Bcl-xL expression. In addition, co-expression of EVI1 with P300/CBP-associated factor (PCAF) abolishes the effect of EVI1 on Bcl-xL, whereas co-expression of EVI1 and the dominant-negative form of PCAF showed no effect on EVI1 activity, suggesting that acetylation of EVI1 abrogated its ability to bind to the Bcl-xL promoter and promote Bcl-xL expression [22]. EVI1 delays cell proliferation and cell cycle progression. Compared to control siRNA, knockdown of EVI1 with EVI1-specific siRNA decreased the number of cells in the G0/G1 phase and increased the number of cells in the S and G2/M phases by 12–14%. They revealed that EVI1 directly binds to the DeltaNp63 promoter element and downregulates its expression. Downregulation of DeltaNp63 promotes p21 expression in HT29 cells and HCT116 cells, as well as in colon cancer patient samples with a low level of p53 [23]. Nayak et al. [24] later analyzed colon cancer patient microarray datasets and observed a negative correlation between EVI1 expression level and several epithelial-mesenchymal transition (EMT)-related markers, including SLUG, ZEB1, ZEB2, SNAIL, TWIST1 and TWIST2. Later, they performed a ChIP assay and luciferase reporter assays and showed that EVI1 directly binds to the SLUG promoter element via the second zinc finger domain and downregulates its expression. Invasion assays revealed that knockdown of EVI1 increased invasion activity and resulted in EMT-like morphological features such as a spindle-shaped appearance with a significant downregulation of the epithelial marker *E*-CADHERIN and upregulation of the mesenchymal marker *N*-CADHERIN in COLO205 cells. Injection of these EMT-induced COLO205 cells into mice failed to show metastasis in any organ, whereas injection of the EVI1-positive non-EMT COLO205 cells showed metastasis in the intraperitoneal layer as well as in the lungs and spleen. These studies demonstrate that EVI1 inhibits EMT by suppressing SLUG transcription. Inhibition of EMT does not abolish the ability of EVI1 to promote tumorigenicity and metastasis in colon cancer [24].

**Figure 3:**
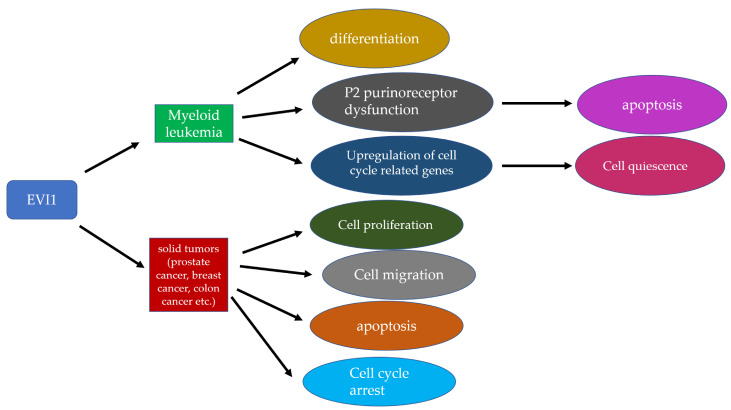
Functions of *EVI1* in leukemia and solid tumors. *EVI1* regulates differentiation, apoptosis and cell quiescence in leukemia and cell proliferation, migration, apoptosis and cell cycle in solid tumors.

In summary, EVI1 and its variants play important functions in leukemia and solid tumors, as summarized in Figure 3.

## 4. Downstream Signaling Pathways

**Figure 4:**
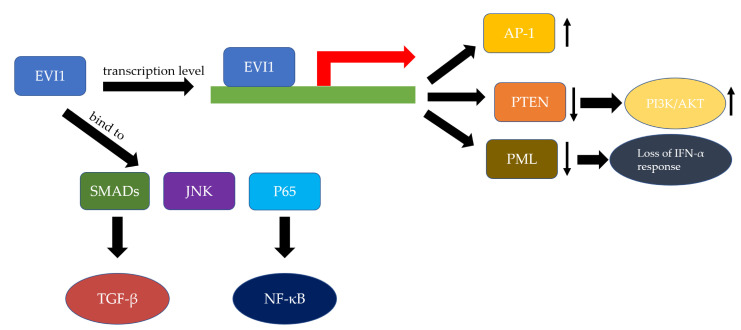
Downstream signaling pathways of *EVI1*. Known downstream signaling pathways of EVI1 include the PTEN/PI3K/AKT, TGF-β signaling, JNK and NF-kB pathways. The green bar represents DNA and the red arrow indicates the transcription start site.

Although the molecular mechanism by which EVI1 promotes leukemia transformation is still unclear, many studies have been conducted in different cell systems to investigate the downstream signaling pathways of EVI1, by which it exerts its biological functions (Figure 4). Studies have shown that EVI1 represses transforming growth factor β (TGF-β) signaling and antagonizes the growth-inhibitory effects of TGF-β. [27]. The transcriptional repression of EVI1 is mediated by the interaction of EVI1 with SMAD3 via zinc fingers 1–7 [27]. Deletions of zinc fingers 1–7 of EVI1 abolish its ability to bind to SMAD3 and to inhibit TGF-β-mediated transactivation activity [27]. In addition, Izutsu et al. [28] demonstrated that TGF-β activation of the reporters is partially impaired by an EVI1 missense mutant which lacks the ability of CtBP binding, suggesting the interaction of EVI1 with CtBP through the EVI1 repression domain is also necessary for the repressive effect [28]. EVI1 has also been shown to interact with other SMADs, including SMAD2 and SMAD4, suggesting that EVI1 may play a regulatory role in signaling mediated by other TGF-β family members such as activin and BMPs [29]. Further studies are needed to determine whether that is the case.

EVI1 represses the transcription of PTEN in murine bone marrow, which results in the activation of the AKT/ mTOR signaling [30]. Knockdown of EVI1 increased the PTEN protein level and decreased phosphorylation of AKT and mTOR [30]. ChIP assays revealed that EVI1 interacts with several polycomb group proteins and recruits polycomb-repressive complexes to the PTEN promoter region, which induces histone modification to repress PTEN transcription. It represents a novel epigenetic mechanism of AKT/mTOR activation in leukemia [30]. These results indicate that the interaction between the PTEN/AKT/mTOR signaling pathway and the EVI1-polycomb complexes could be potential therapeutic targets for leukemia with activated EVI1 [30]. Knockdown of EVI1 also represses cell proliferation and promotes apoptosis via the PTEN/AKT signaling pathway in hilarcholangiocarcinoma [31].

In addition, EVI1 increases AP-1 activity by promoting transactivation of the c-fos promoter [32]. Deletion mutants of EVI1 were constructed to demonstrate that the second zinc finger domain is essential for AP-1 activation and transactivation of the c-fos promoter [32]. Later, Kurokawa et al. [33] showed that blocking of the ERK pathway by a catalytically inactive form of ERK or a dominant-negative form of MEK1 markedly repressed EVI1-induced AP-1 activity, suggesting that ERK signaling is essential for the efficient induction of AP-1 by EVI1.

EVI1 acts as an inhibitor of c-Jun *N*-terminal kinase (JNK), which is a class of mitogen-activated protein kinases implicated in cell stress response [33]. EVI1 physically interacts with JNK via its first zinc finger domain but does not affect JNK phosphorylation [33]. However, this interaction is required for JNK inhibition [33]. In addition, EVI1 protects cells from stress-induced cell death dependent upon its ability to inhibit JNK [33]. These results indicate that EVI1 blocks cell death by selectively inhibiting JNK, thus promoting oncogenic transformation [33].

EVI1 completely abrogates the anti-proliferative and apoptotic effects of IFN-α in hematopoietic cells [34]. EVI1 prolongs STAT1 phosphorylation and binding to the first exon of promyelocytic leukemia protein (PML), thereby repressing PML transcription and preventing the activation of PML-dependent apoptotic pathways, resulting in the loss of IFN-α response [34].

Xu et al. [35] showed that EVI1 negatively regulates the nontypeable *Haemophilus influenzae-* and TNF-α-induced NF-κB activation and subsequent inflammatory responses by inhibiting the DNA-binding activity of the NF-κB complex. EVI1 directly interacts with the NF-κB p65 subunit and inhibits its acetylation at lysine 310, which leads to the inhibition of its DNA-binding activity [35]. In addition, nontypeable *Haemophilus influenzae* and TNF-α can in turn induce the expression of EVI1 in an NF-κB-dependent manner, thereby unveiling a novel inducible negative feedback loop in NF-κB-dependent inflammation [35].

## 5. Conclusions

EVI1 is a 1051 amino acid transcription factor that has been extensively studied due to its association with myeloid leukemia. EVI1 contains two zinc finger domains, a proline-rich repressor domain and a C-terminus acidic domain [38,39]. Both zinc finger domains bind to specific DNA sequences [38,39]. MDS1/EVI1 is encoded by the same locus as EVI1 with the complete sequence of EVI1 and an *N*-terminal extension of 188 amino acids including 125 codons from *MDS1* and 63 codons derived from the second exon and part of the third exon of *EVI1* [99]. The extension region shares homology with the PR domain of RIZ and B-LYMP1 proteins but its function is unclear [44,45,46,47,48]. Previous studies have described EVI1 as a transcriptional repressor, whereas MDS1/EVI1 is a transcriptional activator [4,5].

EVI1 expression is not detectable in the bone marrow of normal individuals. However, in leukemia, *EVI1* transcription has been found to be inappropriately activated in hematopoietic cells [58,100]. Activation of EVI1 transcription is associated with chromosomal rearrangements resulting in either overexpression of the EVI1 protein, or constitutive expression of a fusion protein, AML1/MDS1/EVI1, in which part of AML1 fuses to the *N*-terminus of the entire MDS1/EVI1. AML1/MDS1/EVI1 is a 1395 amino acid protein with an open reading frame of 4185 nucleotides [3]. In AML1/MDS1/EVI1, AML1 is interrupted at the end of the RHD (runt homology domain) [3], followed by the entire MDS1/EVI1 cDNA. Therefore, the AML1/MDS1/EVI1 fusion protein is a chimeric protein which consists of the RHD, PR domain and two zinc finger domains of MDS1/EVI1. AML1/MDS1/EVI1 has a similar function to EVI1 as a transcription factor [6,7]. Studies have also shown that EVI1 and AML1/MDS1/EVI1 both play an important role in leukemogenesis.
